# Lipoxygenases at the Intersection of Infection and Carcinogenesis

**DOI:** 10.3390/ijms25073961

**Published:** 2024-04-02

**Authors:** Abdul-Saleem Amoah, Nikolay B. Pestov, Tatyana V. Korneenko, Igor A. Prokhorenko, Georgy F. Kurakin, Nickolai A. Barlev

**Affiliations:** 1 Institute of Biomedical Chemistry, Moscow 119121, Russia; inst@ibmc.msk.ru (A.-S.A.); nikolai.barlev@nu.edu.kz (N.A.B.); 2 Laboratory of Molecular Oncology, Phystech School of Biological and Medical Physics, Moscow Institute of Physics and Technology, Dolgoprudny 141701, Russia; 3 Group of Cross-Linking Enzymes, Shemyakin-Ovchinnikov Institute of Bioorganic Chemistry, Moscow 117997, Russia; korn@mail.ibch.ru (T.V.K.); prig67@mail.ru (I.A.P.); 4 Laboratory of Tick-Borne Encephalitis and Other Viral Encephalitides, Chumakov Federal Scientific Center for Research and Development of Immune-and-Biological Products, Moscow 108819, Russia; 5 Vavilov Institute of General Genetics, Moscow 119991, Russia; 6 Department of Biochemistry, Pirogov Russian National Research Medical University, Moscow 117513, Russia; kurakin_gf@rsmu.ru

**Keywords:** ALOX, cancer, carcinogenesis, cystic fibrosis, lipoxygenase, inflammation, *Pseudomonas aeruginosa*

## Abstract

The persisting presence of opportunistic pathogens like *Pseudomonas aeruginosa* poses a significant threat to many immunocompromised cancer patients with pulmonary infections. This review highlights the complexity of interactions in the host’s defensive eicosanoid signaling network and its hijacking by pathogenic bacteria to their own advantage. Human lipoxygenases (ALOXs) and their mouse counterparts are integral elements of the innate immune system, mostly operating in the pro-inflammatory mode. Taking into account the indispensable role of inflammation in carcinogenesis, lipoxygenases have counteracting roles in this process. In addition to describing the structure-function of lipoxygenases in this review, we discuss their roles in such critical processes as cancer cell signaling, metastases, death of cancer and immune cells through ferroptosis, as well as the roles of ALOXs in carcinogenesis promoted by pathogenic infections. Finally, we discuss perspectives of novel oncotherapeutic approaches to harness lipoxygenase signaling in tumors.

## 1. Introduction

It is well established that many strains of *Pseudomonas aeruginosa* (*PAE*) produce the non-specific lipoxygenase LoxA, and its homologs are present in some other opportunistic pathogens.

Gene expression studies have demonstrated elevated mRNA expression levels of LoxA during infection and biofilm formation [1]. LoxA contributes to *PAE* pathogenesis in cystic fibrosis patients by triggering lipid peroxidation-dependent cell death—ferroptosis, following the oxidation of host arachidonic acid (AA) containing phosphatidylethanolamines to 15-hydroperoxy-AA-phosphatidylethanolamines in the bronchial epithelial cells of humans [2,3,4].

Pro- and anti-tumorigenic roles of lipoxygenases, especially the immune cell-specific ALOX5 and reticulocyte-type 15-ALOX/ALOX15 have been already discussed in many reviews. Here, we attempt to link these data to the most recent discoveries, asserting that the activation of some sensors of innate immunity can lead to excessive lipid oxidation while switching on the eicosanoid oxidation cascades. Furthermore, we hypothesize that certain ALOXs can have the propensity to play a distinct role in exacerbating the transition from pre-cancerous conditions to aggressive cancers.

## 2. Overview of Molecular Evolution of Lipoxygenases

### 2.1. Structure-Functional Basis of PUFA Lipoxygenation

Arachidonic acid and related polyunsaturated fatty acids (PUFAs) are the precursors of hundreds of signaling lipids that exert various biological functions in a diverse set of organisms, ranging from plants to humans [5]. These biologically active lipids are referred to as oxylipins, which is a common name for oxygenated unsaturated fatty acid derivatives. Twenty-carbon oxylipins are referred to as eicosanoids (from the Ancient Greek word “εἴκοσι” for “twenty”) [6]. Since many human oxylipins have 20 carbon atoms, this name is commonly used in biomedicine. This is the reason why the human oxylipin pathway is usually called the eicosanoid pathway.

The eicosanoid pathway is associated with two unsurprisingly intertwined processes: inflammatory response and cancer. Recent advances in lipidomics have allowed the dissection of this evolutionarily conserved signaling network and the identification of the molecular players responsible for the transmittance of signaling cues in cells. In this review, we focus on lipoxygenases: critical oxidoreductases of the eicosanoid pathway that catalyze the oxygenation of polyunsaturated fatty acids to their corresponding hydroperoxide derivatives and other downstream products. We attempt to highlight the intersecting roles of lipoxygenation in infection and carcinogenesis.

**Figure 1:**
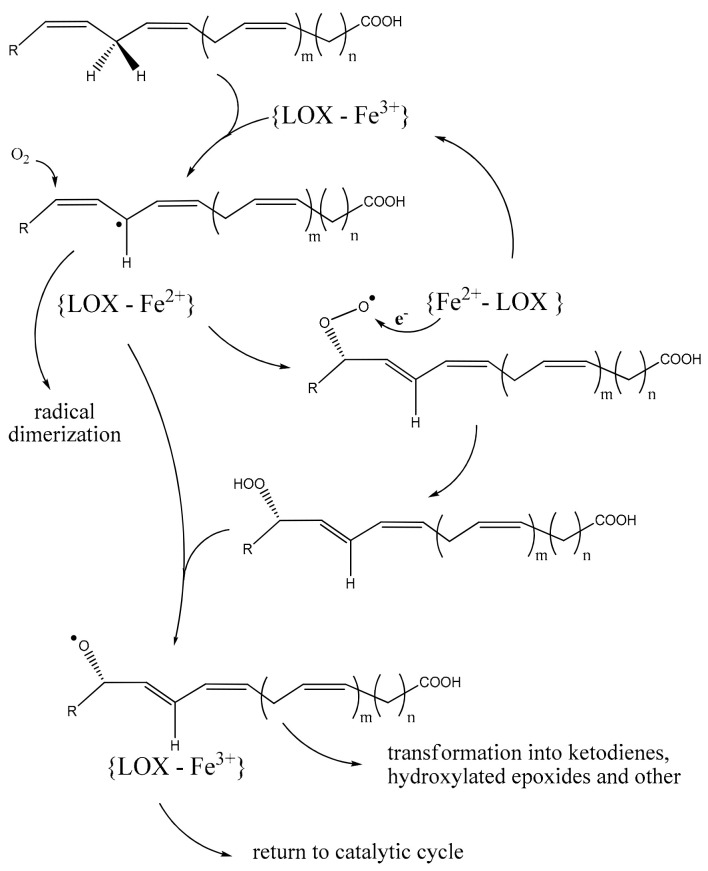
Catalytic cycle of ALOX.

Lipoxygenases are vital players in the deeply rooted evolutionary process of oxylipin signaling, which can be ascribed to essential elements such as the ubiquitous presence of oxygen and polyunsaturated fatty acids (PUFAs) [7]. Lipoxygenases generate PUFA hydroperoxides through four sequential reactions (Figure 1):

A fatty acid radical is formed by removing hydrogen from a bis-allylic methylene. Radical fatty acid undergoes an intermolecular rearrangement. Incorporation of molecular oxygen results in peroxyl radical formation. The peroxyl radical is reduced, converting it into its corresponding anion [8].

Lipoxygenases have a single amino acid chain with a non-heme iron and a Fe-O-H-C bridge. In the 3D structure of rabbit 15-lipoxygenase, iron is coordinated by four histidine side chains and the C-terminal isoleucine [9]. Animal lipoxygenases seem more structurally flexible than their plant counterparts, with evident flexibility between the domains during their cycle. Indeed, compared to soybean lipoxygenase-1, rabbit 15-lipoxygenase has increased structural flexibility, especially in its linker domain [10,11,12].

**Figure 2:**
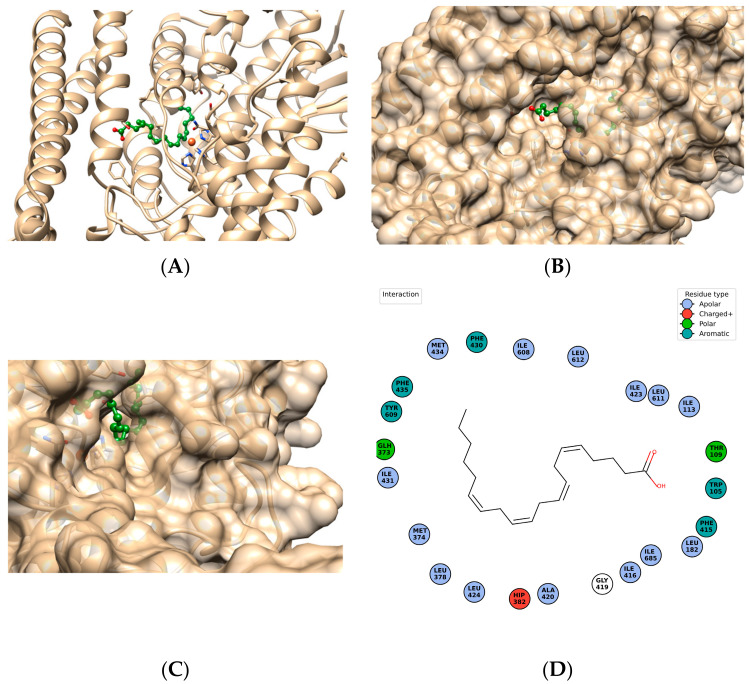
Substrate binding cavity of ALOX. (**A**) The docking of arachidonic acid into the *Pseudomonas aeruginosa* lipoxygenase [14] (PDB ID: 4g33) shows the position and the binding site which is normally occupied by a fatty acid. The fatty acid adopts the curved form of the binding site upon approaching the iron ion, coordinated by histidine residues. (**B**) The binding site has a narrow aperture. (**C**) An additional image to show the curved narrow form of the binding site. (**D**) PlexView 2D interaction diagram shows that the binding site is lined by predominantly apolar and aromatic residues. Docked with AutoDock Tools [15] and AutoDock Vina [16], visualization with UCSF Chimera [17].

The substrate binding pocket in these enzymes resembles an oblongated U-shaped funnel with a dead end and an arrow aperture, which is lined with predominantly hydrophobic residues (Figure 2). While dioxygen follows through a defined path within the enzyme, this channel is not highly conserved. The specific isoform of lipoxygenase is characterized by specific regioselectivity, selecting which carbon atom in the fatty acid is oxygenated. The stereoselectivity of lipoxygenation also depends on the isoform [13].

**Figure 3:**
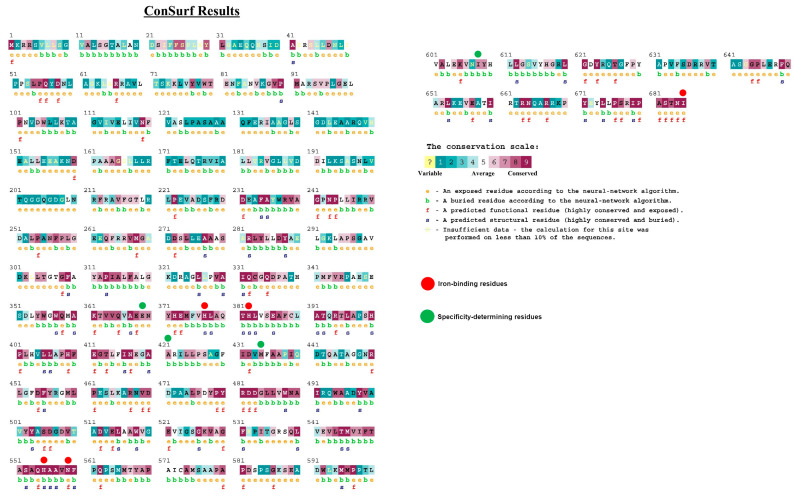
ConSurf output from the alignment of *Pseudomonas aeruginosa* lipoxygenase with some other pathogen and symbiont ALOXs. The metal-binding residues (red circles) are absolutely conserved, while regiospecificity-determining and stereospecificity-determining residues (green circles) are slightly variable. Created with ConSurf [21].

The subsets of amino acid residues defining regio- and stereospecificity have been comprehensively characterized [9,18,19,20]. They can be slightly variable within the same type of lipoxygenases until their physical properties are preserved. The most conserved amino acid residues are metal-binding residues complexing catalytic iron ion (Figure 3).

**Figure 4:**
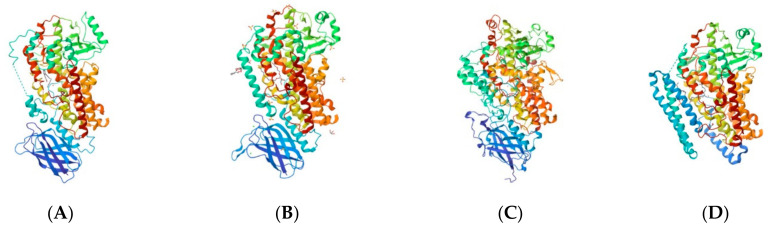
Three-dimensional structures of lipoxygenases. The mammalian lipoxygenases (**A**–**C**) are folded into classical two-domain structure: N-terminal β-sheets depicted in deep blue color and the C-terminal α-helices in varying colors (red, yellow, green). The polypeptide chain of *Pseudomonas aeruginosa* lipoxygenase (**D**) is folded into a single α-helix domain. (**A**): Three-dimensional Structure of Rabbit Reticulocyte 15-Lipoxygenase (PDB ID: 1LOX) [23]. (**B**): Structure of human ALOX15B with a substrate mimic (PDB ID: 4NRE) [22]. (**C**): Structure of a stable ALOX5 (PDB ID: 3O8Y) [24]. (**D**): Wild-type bacterial lipoxygenase from *Pseudomonas aeruginosa* (PDB ID: 5IR5) [25].

Human lipoxygenases exist as a single polypeptide chain folding in two domains: the larger catalytic domain consists predominantly of α-helices defining the active pocket and catalytic properties of the enzyme. The N-terminal is a β-barrel domain featuring β-sheets in both parallel and antiparallel orientations, similar to that of lipases. It makes up approximately 15 kDa and it primarily regulates the membrane binding ability of the enzyme [13]. The crystallized structure of rabbit 15-lipoxygenase has been completely elucidated and serves as an ideal model for other ALOXs including human ALOX5 and ALOX15 [22] (Figure 4). Truncation of the N-terminal domain does not inactivate the enzyme [12].

### 2.2. Plants and Fungi

Initially identified as carotene oxidase, lipoxygenases were first discovered in plants in the early 20th century [10]. Indeed, these enzymes are ubiquitously present in plants and start an oxylipin pathway leading to the synthesis of diverse oxylipins involved in defensive and stress-related reactions including reactions against microbial pathogens. These oxylipins include microbicidal divinyl ethers, volatile oxylipins and potent plant hormones such as jasmonates [11]. Plant lipoxygenases are moreover significantly involved in defense responses against biotic and abiotic factors like viruses, bacteria, fungi, and insects. Lipoxygenase-derived oxylipins are crucial players in the related physiological processes including growth, germination of seeds, ripening of fruits and senescence. [11]. Plant lipoxygenases can be regio-specifically classified into either 9-lipoxygenases or 13-lipoxygenases, with few instances of dual positional specificities (9/13 lipoxygenases) [26]. Most of the metabolites derived from the 9-lipoxygenase pathway (such as 9(S)-keto octadecatrienoic acid) regulate defense responses against pathogens. Mechanistically, 9-hydroxyoctadecatrienoic acid induces cell wall modifications by inhibiting cellulose synthesis to restrict the invasion of *Pseudomonas syringae* in Arabidopsis [27]. 9-hydroxyoctadecatrienoic acid has also been shown to be a potent inducer of root waving [28]. The 13-lipoxygenase pathway leads to the synthesis of plant signaling compounds like jasmonates and green leaf volatiles. [11] Plant lipoxygenases have been successfully isolated from many plants such as *Zea mays*, *Oryza sativa*, *Capsicum annuum* and *Arabidopsis thaliana* [10].

In fungi, the most interesting aspect of lipoxygenase function in the context of the current review is the putative involvement of lipoxygenase-derived oxylipins. *Candida albicans* is reported to synthesize resolvin E1 (an anti-inflammatory oxylipin) probably via a lipoxygenase-dependent pathway. This suggests its possible role in immune crosstalk between the *C. albicans* and the host. *Gaeoumannomyces graminis* uses lipoxygenase to penetrate host tissues. Besides pathogenicity, lipoxygenases in fungi are involved in morphological shifts and quorum sensing processes; this is highly suggestive for their role in intra-species cell-to-cell signaling, which is similar to the case of mammals and plants [29].

It is also worth mentioning that some fungi such as *Botrydiplodia theobromae*, *Lasiodiplodia theobromae*, *Aspergillus niger* and *Fusarium oxysporum* are able to synthesize jasmonate analogues [29]. Despite the fact that their biosynthesis pathway is still poorly characterized, their possible role is identified as mimicking the natural plant’s jasmonate signaling to hijack it and downregulate the plant’s immunity. This, in turn, facilitates invasion. This biochemical peculiarity is highly suggestive that some human bacterial pathogens may exert a similar process in opportunistic infection, including patients with cancer and other malignant comorbidities.

### 2.3. Prokaryotes

Besides plants, lipoxygenases have also been widely discovered in mammals [30] and to a lesser extent, bacteria [31]. Microbial lipoxygenases have not received much attention in research, with the majority of studies limited to eukaryotic organisms for the most part [32]. There is ongoing debate about whether the numerous lipoxygenase sequences found in databases of microbes like *PAE* are due to sampling biases. With only a handful of exceptions, bacteria with putative lipoxygenase-encoding genes are expected to make up not more than 0.5% of all sequenced bacteria. These bacteria belong to the phyla Cyanobacteria and Protobacteria. This insufficient gene distribution in bacteria implies lipoxygenase-encoding genes could potentially be acquired through horizontal gene transfer [33,34]. Given the total number of sequenced bacterial genomes, this results in a few hundred putative bacterial sequences, but we estimate the number of biochemically characterized lipoxygenases to be in the order of tens, based on a recent review by Chrisnasari et al. [18] and given the growing number of routine biochemical analyses of lipoxygenases for the purposes of biotechnology. However, biotechnological papers provide almost no data about the biological function of prokaryotic ALOXs in natural settings. Such experimental data are restricted to single works on *Myxococcus xanthus* and *Nostoc punctiforme*, as well as a few works on *PAE* lipoxygenase (which will be discussed in the next section). Ultrasound-induced injury in *N. punctiforme* releases lipoxygenase-derived oxylipins—but the reactions they are expected to elicit remain obscure [35].

Attempts to recompense for this lack of experimental data with a bioinformatic study first led to finding a statistical and phylogenetic link between lipoxygenases and multicellularity in bacteria, and secondly led to the hypothesis that bacterial oxylipins are involved in forming and maintaining multicellular structures [36,37]. However, the definitive confirmation of this hypothesis has not yet been obtained. Moreover, these works revealed the second association between bacterial lipoxygenases and opportunistic pathogenicity, which will be discussed in detail in later sections.

Even though experimental data are scarce, bioinformatic analysis identified lipoxygenases in a range of opportunistic pathogens, such as *PAE*, *Burkholderia gladioli*, *Nocardia brasilensis*, *Nocardia pseudobrasilensis*, and some other pathogens [37,38]. Bacterial lipoxygenases have been found to act on a wide range of PUFAs such as arachidonic, linoleic, eicosapentaenoic and docosahexaenoic acids. PUFAs were not detected in *Burkholderia thailandensis* [39]. Interestingly, lipoxygenase from this pathogen has been shown to produce 15-HETE from arachidonic acid, which is further metabolized into bioactive lipid compounds including lipoxins and leukotrienes [40]. Identical to *PAE*, *B. thailandensis* can regulate host immune defense through alteration of local immune mediators. *B. thailandensis*, which is also closely related to *B. gladioli*, has been successfully analyzed after its expression and purification. This native enzyme appears as a 150-kDa dimer, with each chain weighing 75 kDa. It exhibited the highest enzymatic activity and catalytic efficiency for linoleic acid, similar to plant ALOXs producing 13-(S)-hydroperoxy-octadecadienoic acid (13-S-HPODE). However, the k_cat_/k_m_ was only two times lower for arachidonic acid and thus the profile for substrate specificity is not very different from that of human ALOXs. Conditions optimized for its activity are pH 7.5 and 25 °C with specific concentrations of linoleic acid. Curiously, the enzyme is stimulated by high concentrations of Cu^2+^ and methanol. Notably, this enzyme’s efficiency surpasses that of commercial soybean lipoxygenase when tested under optimal conditions for each enzyme at similar substrate and enzyme concentrations [41]. The kinetic variables of various lipoxygenase isoforms including that of soybean are detailed in Table 1.

Pathogens such as *PAE* and *B. gladioli* can employ lipoxygenases to counteract anti-pathogen defenses in plants, facilitating pathogenic transition from plants to humans—a phenomenon known as interkingdom pathogen transfer [38].

### 2.4. Lipoxygenase (LoxA) in Pseudomonas aeruginosa

Amongst the well-studied bacterial lipoxygenases is LoxA, first characterized in 2004 from the opportunistic pathogen *PAE* [56]. *PAE* is a highly versatile Gram-negative opportunistic pathogen, dominant in patients with chronic lung infections, particularly in cystic fibrosis patients and immunocompromised cancer patients [56,57]. The pathogen’s capacity to invade host tissues and circumvent immune defenses is attributed to virulence factors secreted via types 2 and 3 secretion systems [57,58]. These secretions comprise an arsenal of enzymes involved in host lipid metabolism that facilitates the pathogen’s environmental adaptation and immune system modulation, leading to the exacerbation of infections [59,60,61].

Extensive in vitro and in vivo biochemical studies have characterized a highly conserved and secretable LoxA enzyme [4,56] capable of oxidizing a wide range of free PUFAs including omega 6 and omega 3 fatty acids during chronic lung infection in cystic fibrosis patients [59]. Contrary to mammalian lipoxygenases, LoxA carries a unique N-terminal signal sequence that earmarks it for secretion predominantly to the periplasmic space, with a fraction of the active protein further secreted to the extracellular milieu in a fashion dictated by the Xcp type II secretion apparatus [56]. It is noteworthy that a periplasmic enzyme like LoxA has the ability to convert exogenous substrates for extracellular release due to the molecular masses of arachidonic acid and other PUFAsubstrates (304.5–320.5 Da) which are small enough to transit through *PAE*s outer membrane. Evidently, the treatment of intact bacteria with arachidonic acid produced a significant fraction of cell-free 15-HETE, unconfined in the periplasm of *PAE* [56]. Thus, this finding strongly supports studies demonstrating that some strains of *PAE* make available to themselves pools of free host PUFA substrates by secreting a specific phospholipase ExoU cytotoxin, to trigger the release of PUFAs from host cell membranes [43,62]. Considering its apparent suitability to metabolize exogenous substrates, LoxA happens to proficiently act on host substrates in order to potentially modify local inflammatory signals during infections. These data suggest a significant biological role for LoxA in modulating host-pathogen interactions. Numerous studies have identified LoxA enzyme as an exhibitor of ALOX15 activity, localization in the periplasm [56], and a capability to oxidize various free and membrane-bound polyunsaturated fatty acids (such as arachidonic, linoleic, oleic, and linolenic acids) to produce ALOX15 derivatives [32]. Despite its functionality, LoxA displays limited sequence similarity to human ALOX15 (39%) and soybean ALOX15 (37%) [43].

### 2.5. Bacterial Lipoxygenases, Cystic Fibrosis and Malignant Conditions

Recent bioinformatic studies have identified the link between the presence of lipoxygenase sequences in bacteria and their ability to cause lung infection in cystic fibrosis patients [38]. In the data analysis by Kurakin (2022) [38], “cystic fibrosis” was specially highlighted as the term with a huge connectivity to other nodes on the graph model. This underscores that lung damage reflects some intrinsic property of bacterial lipoxygenases.

Cystic fibrosis (CF) is a genetic disorder resulting from mutations in the cystic fibrosis transmembrane conductance regulator (*CFTR*) gene that affect the digestive system and respiratory tracts. Defects in this gene disrupt the formation of ion channels responsible for the transport of water and ions (chloride and bicarbonate ions) in exocrine and sweat glands, causing a build-up of highly viscous sticky mucus which clogs up vital organs not limited to the lungs and pancreas [63]. This obstructs airways and enhances colonization by pathogens. Inflammatory response to infections sets off a vicious loop leading to the failure of the respiratory systems and surrounding organs [64]. Common symptoms of cystic fibrosis include malnutrition and poor growth due to obstructions in both the transport of digestive enzymes from the pancreas and food breakdown in the intestines. Breathing abnormalities and persistent cough due to the blockage of airways in the lungs are other resulting indicators.

There are diverse molecular mechanisms explaining the cause of CF, although the consequences of most mutations have been associated with a decrease or loss in CFTR protein function. A number of mutations have an effect on the production of CFTR mRNA—influencing the length or amount of mRNA produced. These comprise splicing mutations, significant insertions and deletions, and nonsense mutations [64]. Mutation F508del interferes with the intramolecular interactions of CFTR protein leading to delays in folding processes, trafficking, and the facilitation of premature chaperone-assisted degradation of the misfolded protein. Structural molecular changes in the protein have been attributed to long exposure of hydrophobic regions to the aqueous environment which eventually disrupts protein-protein interruptions [65]. Apart from F508del, a variety of mutations are believed to induce CTFR misfolding, however the disruption of intramolecular interactions may vary depending on the location of mutations [66]. Specific mutations in positions that build up the anion pore have also been described to solely affect protein channel function by impairing conductance via unbalanced anion selectivity [67].

*PAE* is undoubtedly one of the prevalent pathogens that thrives and aggravates conditions in the lungs of CF patients. Therefore, the available experimental data on the role of bacterial lipoxygenase in lung damage regard only this species. As the preferential free substrate of LoxA, DHA and other PUFAs present in the human lung mucosa are metabolized into specific ALOX15 metabolites including 17-hydroxydocosahexenoic acid (17-HdoHE) and 15-HETE, ultimately triggering the production of anti-inflammatory and pro-resolving lipid mediator LXA4 [59]. It is noteworthy that LXA4 resolves acute inflammation even at nanomolar concentrations. The biosynthesis of this pro-resolving lipid requires transcellular mechanisms involving several cell types (leukocytes, endothelium, and epithelium) together with the coordinated action of other ALOXs (ALOX5). The overproduction of LoxA-dependent products like LXA4 downregulates major chemokines (MIP-2/CXCL-2, KC/CXCL-1) and limits the recruitment and chemotaxis of leukotrienes and leukocytes critical for inflammatory response and antimicrobial defense via a paracrine or autocrine axis [59,68]. Consequently, LoxA minimizes *PAE* clearance in the lung, thus conferring a survival advantage to the Gram-negative opportunistic pathogen [59]. Even more interesting, a similar study described the sidetracking of host immune response via marked production of AA-derived LXA4 by a specific ALOX15 from apicomplexan protozoan, *Toxoplasma gondii* [69]. Mechanistically, *Toxoplasma gondii*-derived-LXA4 suppresses interleukin-12 production by dendritic cells, delaying early innate immune response and augmenting *Toxoplasmic encephaliti* [69]. These data underscores that the production of pro-resolving lipid mediators can be the general scheme by which pathogens modulate host-pathogen interaction.

LoxA is not the only agent in *PAE* which facilitates the colonization of the pathogen in the airways of immunocompromised patients. *PAE* has been shown to secrete a virulence factor known as cystic fibrosis transmembrane conductance regulator inhibitory factor (Cif)—an epoxide hydrolase that disrupts endocytic recycling of CFTR, consequently minimizing the abundance of CFTR in host epithelial membranes [70]. Hvorecny and colleagues firmly postulated that Cif-mediated ion depletion minimizes mucociliary transport which ultimately hinders bacterial clearance from the lungs. Aware of the fact that an inverse correlation exists in the airways between Cif and a host defensive pro-resolving lipid (15-epi-lipoxin A4), the same study reported upregulated levels of 15-epi-lipoxin A4 in murine lungs infected with a *PAE* strain expressing inactive Cif relative to those infected with wild-type *PAE* [70]. These findings stress the acknowledgement of Cif as a virulent factor in *PAE* pathogenesis in immunocompromised patients.

The example of plant-fungal jasmonate signaling in pathogenesis (mentioned above) makes this hypothesis even more plausible.

Bioinformatics studies suggest that not only *PAE*, but also other pathogens like *B. gladioli*, *N. brasiliensis*, and *Pluralibacter gergoviae* might employ lipoxygenases to counteract plant anti-pathogen defenses, facilitating their transition from plants to humans—a phenomenon known as interkingdom pathogen transfer [37,38].

Regarding the oncological perspective, bacterial lipoxygenases might be associated with such a group of malignant conditions as leukemia. The term “leukemia” is present in two bacterial species in the data analysis on bacterial lipoxygenases in opportunistic pathogenicity [38]. Leukemia is often treated by chemotherapy which causes a serious depletion of immune cells and renders patients immunocompromised. Given this fact, it is plausible that lipoxygenase-positive bacteria could pose a threat to leukemia patients on the public health scale.

## 3. Inventory of Human and Mouse Lipoxygenase Isoforms

In 1974, the discovery of a 12-lipoxygenase enzyme in human platelets led to its subsequent identification as the platelet-type 12-lipoxygenase, encoded by the *ALOX12* gene. Another isoform known as the reticulocyte-type 15-lipoxygenase (ALOX15) was later found in the lysate of immature red blood cells, playing a primary role in the oxidation of its membrane lipids. Over time, various isoforms of this enzyme have been discovered, each bearing unique characteristics. Following the comprehensive sequencing of the human genome, six distinct functional lipoxygenase isoform genes were identified: *ALOX15*, *ALOX15B*, *ALOX12*, *ALOX12B*, *ALOX5*, and *ALOXE3* [71,72].

### 3.1. ALOX12

Also known as “platelet-type”, ALOX12 was the first mammalian lipoxygenase to be characterized when it was discovered in human platelets in 1974. It incorporates molecular oxygen at C12 of arachidonic acid to produce 12(S)-hydroperoxy-5,8,10,14-eicosatetraenoic acid (12(S)-HpETE) and encodes a 663-amino-acid residue protein with a molecular mass of 75 kDa [8]. Apart from being expressed in platelets and megakaryocyte cells, ALOX12 was recognized in germinal layer keratinocytes. It is abundant in the settings of lung carcinoma, epidermoid carcinoma, and psoriasis [73].

Determining the function of this enzyme in human and mouse platelets has been particularly challenging. Creation of *ALOX12* knockout mice did not uncover convincing roles besides a minor effect in regulating ADP-induced aggregation of platelets [74]. Nevertheless, ALOX12 has been suggested to somewhat maintain water permeability of the skin [75]. Besides *ALOX12*, the normal mouse skin expresses *ALOX12B*, *ALOXE3* and *ALOX15B*. Knockout of *ALOX12* in mice is viable and it reproduces normally [74]; thus, the exact biological role of this enzyme is not completely understood and the defective function of *ALOX12* is possibly compensated for by other lipoxygenase isoforms.

### 3.2. ALOX12B and ALOXE3

12(R)-hydroperoxyeicosatetraenoic acid (12(R)-HpETE) producing lipoxygenase (ALOX12B) was the first mammalian ALOX discovered to incorporate molecular oxygen in the R-stereo-configuration. Both mouse and human *ALOX12B* encode proteins with 701 residues, sharing 86% identity [76]. Even though both orthologs show weak activity on arachidonic acid—the 12-hydroxyeicosatetraenoic acid product generated is of the R-stereo configuration [77]. The mechanism for preferentially inserting oxygen in the R-configuration was later established to take place in an ordinary glycine residue close to the active site [78]. It is specifically expressed in the suprabasal keratinocytes of the skin and hair follicles [77]. A potential biochemical route for regulating the epidermal barrier has been put forward [79]. Here, ALOX12B converts linoleoyl-ω-hydroxyceramide in the stratum corneum to 9(R)-hydroperoxylinoleoyl-ω-hydroxyceramide. Further activity by ALOXE3 (see next paragraph), followed by cleavage of the fatty acyl moiety makes room for conjugation of ω-hydroxyceramide with proteins, forming protein-lipid scaffolds which sustain and support the permeability barrier of the epidermis.

**Figure 5:**
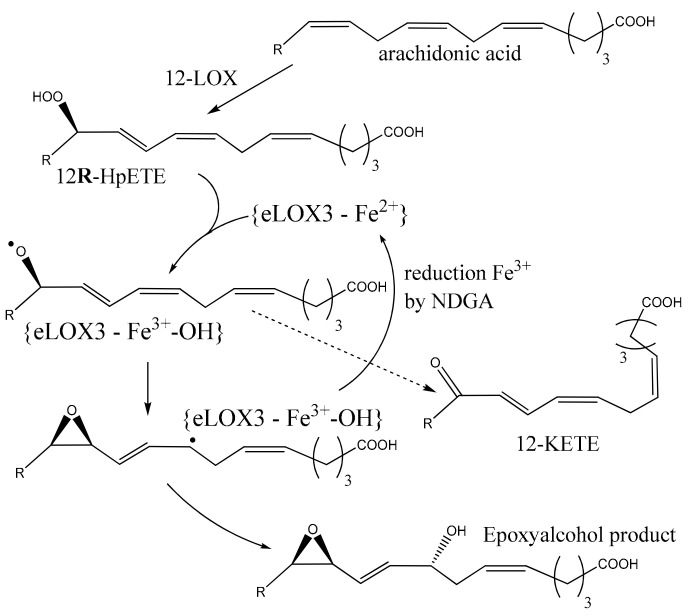
Reaction catalyzed in the skin by the ALOX12B and ALOXE3 ensemble.

Mouse *ALOXE3* encodes an epidermis-type lipoxygenase-3 of 711 amino acids exhibiting 54% sequence identity with ALOX12B. It is present in the epithelia of the skin, forestomach and tongue [8]. ALOXE3 lacks classical oxygenase activity and no enzymatic activity is observed following incubation with arachidonic acid under standard conditions [80]. As an epoxyalcohol synthase, ALOX3E utilizes 12(R)-HpETE as preferred substrate, converting it less efficiently to other hydroperoxides, and more efficiently to 12-ketoeicosatetraenoic acid (12-KETE) and hepoxilin A3 isomer (8(R)-hydroxy-11(R),12(R)-epoxyeicosatrienoic acid) in a 1:2 ratio (Figure 5). Consequently, ALOXE3 was identified as a hydroperoxide isomerase which incorporates two oxygen atoms from 12(R)-HpETE into the epoxyalcohol, and is hypothesized to be in the ferrous form, dissimilar to other lipoxygenases [81]. The substrate specificities of human and mouse ALOXE3 vary to an extent: while both orthologs prefer substrates of R-stereochemistry, human ALOXE3 has higher affinity for 12(R)-HpETE, whereas the mouse enzyme favorably metabolizes 8(R)-HpETE [82]. Genetic evidence has linked missense and splice site mutations in *ALOXE3* and *ALOX12B* to the incidence of autosomal recessive congenital ichthyosis (ARCI)—a severe disorder of keratinization [83,84]. Clearly, ALOXE3 competes with glutathione peroxidase (GPX) for hydroperoxyl-PUFA derivatives in the keratinocytes. Simultaneous ectopic expression of *ALOXE3* and *GPX* in other tissues may have detrimental effects. Even though it may be experimentally demanding, the combination of *ALOX12B* and *ALOXE3* can be a good transgene combination for cancer gene therapy.

### 3.3. ALOX15

The human *ALOX15* gene encodes 662 amino acids and contains 92 non-synonymous coding sequence variations along with 8 nonsense mutations [85,86]. It is expressed in many epithelial tissues [87] and abundant in organs such as the breast, prostate, skin, lung, esophagus, vagina, and cervix. However, its expression is generally limited in certain epithelial or glandular tissues like the pancreas, duodenum, gallbladder, kidney, and urinary bladder. Tumor environments exhibit fluctuating expressions of *ALOX15* across a broad range of tissues: while it is abundantly present in cancerous tissues of the breast, prostate, lung, head and neck, and colorectal cancers, its expression is lost in tumors of the brain, muscle, or germline cells [88].

The human ALOX15 enzyme was identified to catalyze the S-stereospecific oxygenation of carbon-15 in arachidonic acid, producing 15(S)-hydroperoxyeicosatetraenoic acid, which is subsequently reduced to the more stable 15(S)-hydroxyeicosatetraenoic acid. This enzyme was thus named arachidonate 15-lipoxygenase or 15-lipoxygenase-1.

**Figure 6:**
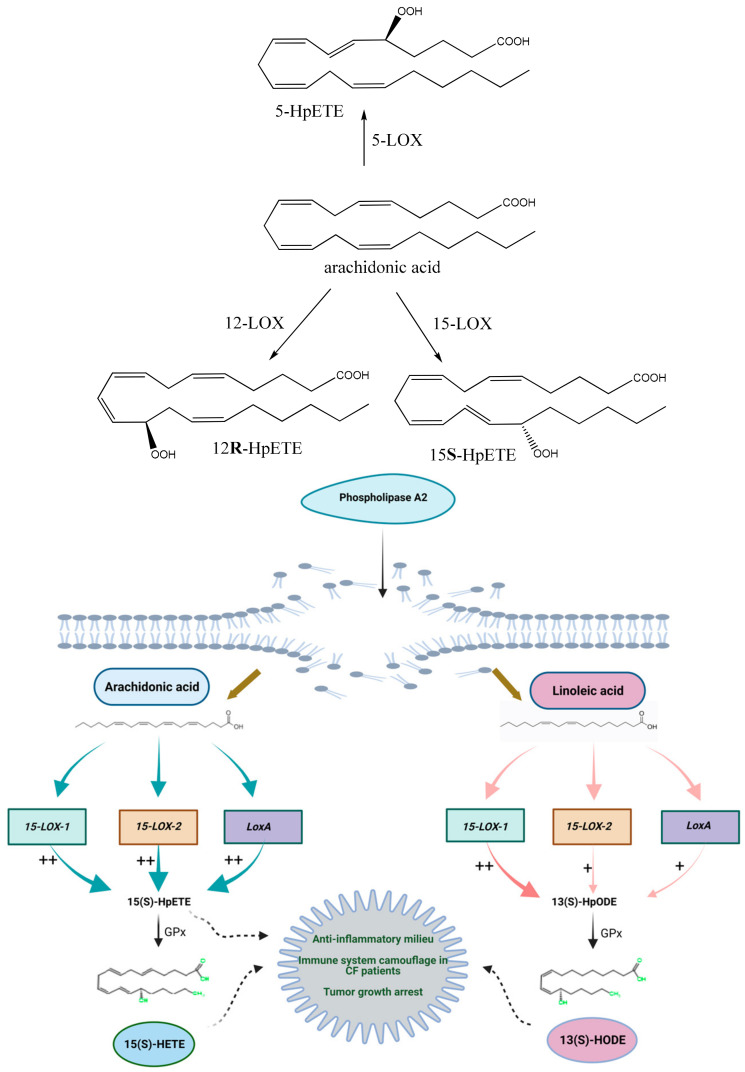
Top panel—Reactions catalyzed by major human ALOXs. Bottom panel—Schematic representation of the metabolism of free membrane fatty acids by 15-LOX isoforms in human (ALOX15, ALOX15B) and *PAE* (LoxA). + is indicative of a low product yield, ++ is indicative of significant product yield. Membrane bound-ω-3 polyunsaturated fatty acids—arachidonic acid and linoleic acid—are hydrolyzed into their unbound forms by the action of phospholipase A2. All three enzymes oxygenate free arachidonic acid with similar affinity to produce 15 hydroperoxyeicosatetratenoic acid (15-HpETE) as a main intermediate. Free linoleic acid is also oxygenated to form 13-hydroperoxyoctadecadienoic acid (HpODE) as a major metabolite of human ALOX15 and a minor metabolite of human ALOX15B and LoxA. Further oxidation by glutathione peroxidase (GPx) leads to the formation of 15-hydroxyeicosatetraenoic acid (15-HETE) and 13-hydroxyoctadecadienoic acid (13-HODE) respectively. 15(S)-HpETE, 15(S)-HETE and 13(S)-HODE are involved in downstream signaling cascades that regulate critical biochemical processes including the deceptive formation of anti-inflammatory environment in *PAE* infections, and growth arrest in some tumors.

ALOX15 predominantly metabolizes PUFAs at the n-6 position, necessitating the initial hydrogen removal from the n-8 carbon atom. Through this mechanism, arachidonic acid undergoes oxygenation at carbon-15, resulting in the formation of 15-hydroperoxyeicosatetraenoic acid (15S-HpETE) [89]. Linoleic acid undergoes a similar chemical modification to produce 13-hydroperoxyoctadecadienoic acid (13-HpODE) (Figure 6). Accounting for roughly 3–15% of the product yield is arachidonic acid-derived 12-hydroperoxyeicosatetraenoic acid (12S-HpETE). This unique characteristic is known as the dual positional specificity of ALOX15 and has been validated in both humans and orangutans [90].

### 3.4. ALOX15B

Human and mouse ALOX15B orthologs display distinct reaction specificities leading to varied reaction products. Targeted double inverse substitution mutations in human *ALOX15B* have been demonstrated to yield products characteristic of mouse metabolism, and vice versa. Ordinarily, the wild-type human ALOX15B enzyme converts arachidonic, docosahexaenoic, and eicosapentaenoic acids into their corresponding hydroperoxyl derivatives. Introducing a double mutation (D602Y + V603H) in this enzyme, however, results in a product pattern unique to mice. In a similar fashion, applying an inverse mutagenesis strategy to mouse ALOX15B (Y603D + H603V exchange) produces reaction products similar to those in human metabolism. This is true for arachidonic and eicosapentaenoic acids, but not for docosahexaenoic acid. With linoleic acid as substrate, the Y603D plus H603V exchange in mouse ALOX15B leads to a human-like product pattern [91]. However, inverse mutagenesis in human ALOX15B creates a racemic product yielding enzyme. This illustrates that specific amino acid substitutions in human and mouse ALOX15B orthologs can convert C-20 fatty acids into murinized or humanized product patterns respectively, but this trend does not consistently apply to fatty acid substrates with varying chain lengths [91].

### 3.5. ALOX12E

Murine epidermal lipoxygenase (*ALOXE*) encodes a 12-lipoxygenase isoform which synthesizes 12(S)-HETE from arachidonic acid and is primarily detected in the mouse epidermis. Sequence homology data show 60% sequence identity in relation to both platelet-type and leukocyte-type murine 12-lipoxygenases. It is however more analogous to platelet-type enzymes with respect to the substrate and product specificities [92]. Both ALOX12E and ALOX12B have been shown to efficiently metabolize fatty acid methyl esters of both arachidonic and linoleic acids relative to the corresponding free substrates. Moreover, both enzymes oxygenated docosahexaenoic acid to respectively 13-hydroxy-docosahexaenoic and 14-hydroxy-docosahexaenoic acids, respectively, independent of calcium concentration while catalytic activities decreasing under acidic pH [93]. MafB is a transcription factor that regulates the differentiation of keratinocytes in both mice and humans. Transcriptional profiling studies demonstrate upregulated expression of lipid-metabolism associated genes like ALOX12E in MafB-deficient mice [94].

### 3.6. ALOX5 and ALOX5AP

ALOX5 is the central player in the initial stages of leukotriene synthesis in which an unstable LTA4 intermediate is produced from arachidonic acid [8]. *ALOX5* is primarily expressed in bone marrow-derived cells including B lymphocytes, dendritic cells, granulocytes, macrophages, and mast cells. It is abundant in organs such as the spleen, intestines, and lungs [8]. High expression levels of *ALOX5*, indicative of abundant macrophage infiltration, have been identified in tumor samples of gastric [95] thyroid [96], pancreatic [97], renal [98], endometrial, and urothelial cancers [99].

The discovery of the inhibitory action of MK-886 on the synthesis of leukotrienes in intact cells led to the discovery of five lipoxygenase-activating protein (FLAP)—a small integral membrane protein essential for ALOX5 activity [100]. Following affinity chromatography-based purification and characterization, the extrapolated amino acid sequence revealed a unique 162 amino acid protein having three proposed transmembrane domains [101]. Transfection of osteosarcoma cells with ALOX5 cDNA alone, and combined with FLAP cDNA show that the small integral membrane protein is critical for the biosynthesis of leukotriene in intact cells [101]. Genetic analysis proves that despite the fact that FLAP-deficient mice breed normally and seem to have no extra inflammatory roles beyond its definitive prerequisite for leukotriene biosynthesis, FLAP-deficient mice exhibit reduced production of 12-HETE and are incapable of metabolizing arachidonic acid to leukotriene products [102].

### 3.7. ALOXes and Esterified PUFAs

The amino-terminal polycystin-1-lipoxygenase α-toxin (PLAT) domain of ALOX15B facilitates transient translocation and binding of the enzyme to membranes. In resting cells, western blotting of cytosol and membrane fractions of ALOX15B-expressing cells show a predominant location of the enzyme in the cytosol [103]. The enzyme shows a preference for arachidonic acid (AA) over linoleic acid (LA) (Figure 6) and typically produces 15-S-HETE, although this specificity might slightly vary between in vivo and in vitro environments [85]

Although 15-lipoxygenase orthologues have been implicated in the production of anti-inflammatory mediators including arachidonic acid-derived lipoxins and specialized pro-resolving lipid mediators (SMP), there is still uncertainty about whether ALOX15B acts on 5-HpETE and if the enzyme is really involved in the generation of lipoxins. In transcellular synthesis events, generation of lipoxin intermediate 5,15-dihydroperoxyeicosatetraenoic acid (5,15-diHpETE) is mediated by ALOX5 oxygenation. Mechanistically, it has been demonstrated that ALOX15B also generates 5,15-diHpETE via oxygenation of 5-HpETE and 5-HETE, but cannot produce lipoxin B4 from 5-HpETE or 5,15-diHpETE because of its inability to remove a hydrogen atom at C10 [104]. Nevertheless, ALOX15B-derived-15-HpETE can serve as a ALOX5 substrate to generate lipoxins [105]. Furthermore, ALOX15B has the ability to transform ALOX5-derived pro-inflammatory mediators into pro-resolving precursors (lipoxin A4) [104]. This counteraction between ALOX15B and ALOX5 indicates an essential switch between pro- and anti-inflammatory precursors, as evidenced in studies of cystic fibrosis macrophages [106] and prostate cancer cells [107]. A case in point is, 5-HETE, which is primarily involved in the proliferation of prostate cancer [108], and overexpression of *ALOX15B* leads to reduced proliferation of prostate cancer cells [107]. This confirms the ability of ALOX15B to class-switch ALOX5 pro-inflammatory products into anti-inflammatory counterparts.

## 4. Normal Function of Lipoxygenation in Human Metabolism and Immunity and Their Weaknesses

**Figure 7:**
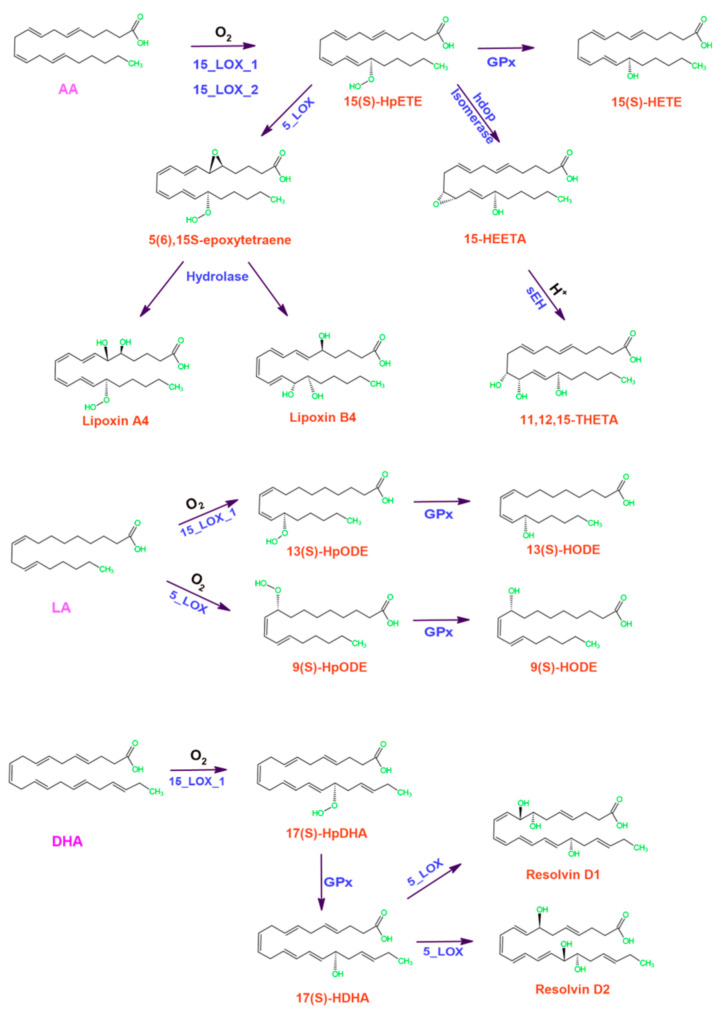
Oxylipins in human immunity. LTB4 drives chemotaxis by binding to the BLT1 receptor, notably on subsets of CD4 and CD8 T cells, however it is active on neutrophils, and undergoes further transformations, thanks to the ALOX5 enzyme and subsequent LXA4 hydrolase actions. 12S-HETE acts as a chemoattractant for neutrophils. AEA: Demonstrating immunosuppressive properties, AEA hinders the migration of CD8 T cells. EPA might serve as a replacement for COX inhibitors. 15-HETE amplifies cell proliferation and boosts the Th1 immune response. 12-HETE is implicated in metabolic adjustments. LXA4: Resulting from ALOX12 action on 5-HETE, LXA4 acts to mitigate inflammation, particularly in adipose tissue, leading to a reduction in IL-6 levels. CYP hydrolases produce 20-HETE, which amplifies inflammatory cytokines and promotes cell adhesion. CYP Epoxygenases: These yield anti-inflammatory epoxides.

Lipoxygenases play pivotal roles in human metabolism and immunity by participating in the biosynthesis of a specific group of oxylipins, which include leukotrienes, eoxins, resolvins, lipoxins, hepoxilins, maresins, and have the dual capacity to both promote and resolve inflammation [8,109,110] (Figure 7).

Inflammation is the immune system’s protective response against deleterious stimuli like pathogens, dead cells, irradiation and toxic substances [111]. During inflammation resolution, the gradual decline of pro-inflammatory signals over time made room for the recognition of the process as a passive event [112]. Recent insights have however retuned this perspective: cessation of acute inflammation is now recognized as an active and tightly regulated complex process driven by changes in inflammatory cues and cellular composition, ultimately restoring normal tissue homeostasis [112]. The processes involved are governed by a balanced interplay of cellular and humoral components from both the innate and adaptive immune systems [13]. There are diverse classes of lipid and non-lipid mediators modulating inflammation. Among lipoxygenase isoforms, ALOX5 exert indisputable functions in human diseases by virtue of its principal role in the synthesis of leukotrienes [113]. Leukotrienes are paracrine pro-inflammatory mediators produced in many leukocytes, particularly in macrophages, dendritic cells and granulocytes; normally functioning in local cellular environments [8]. Leukotriene biosynthesis begins with a classical lipoxygenation at C5 of arachidonic acid forming 5-HpETE, followed by the generation of leukotriene A4 (LTA4) via a process identified as pseudolipoxygenation [8].

Even within low nanomolar levels, leukotrienes are capable of eliciting a plethora of immune regulatory responses. Cysteinyl-leukotrienes, for example, are three orders of magnitude more potent than histamine in inducing the constriction of smooth muscles in the airways [114]. Because of this, ALOX5 and the leukotrienes have been consistently involved in the pathogenesis in several acute and chronic inflammatory human diseases like cystic fibrosis, asthma and cancer [8]. Leukotriene B4—a downstream product of ALOX5—provokes growth of pancreatic tumors through activation of its receptors (BLT1/2), which are as well overexpressed in pancreatic tumor tissues [115]. Interactions between pro-inflammatory mediators and G-coupled proteins on the membranes of immune competent cells leads to a wide range of resolution activities including death of neutrophils by apoptosis, restoration of the permeability and integrity of vascular walls and reduction in migration of leukotrienes to the site of infection [116].

In tissues with high levels of ALOX15B, expression of the enzyme is partly dictated by certain key immune players. Their expression in macrophages is enhanced by lipopolysaccharides (LPS), and interleukins 4, and 13 [117]. In immortalized human keratinocyte cells (HaCaT), *ALOX15B* expression is induced by either interferon-γ or TNF-α [85,86].

## 5. Pre-Malignant Conditions and Benign Outgrowth—Roles of Lipoxygenation

A plethora of lipoxygenase functions have been described in the setting of complex carcinogenesis—but the understudied roles of these enzymes in pre-cancerous milieu leaves a significant gap in fully comprehending mechanics in cancer progression from the onset. A classical case in point is the multiplex inception of precursor lesions in the development of pancreatic cancer—an aggressive cancer with a perplexing prognosis [118]. The progression from pancreatic intraepithelial neoplasia (PanIN) to pancreatic ductal adenocarcinoma (PDAC) is not straightforward: although oncogenic K-RAS mutations is a critical hallmark in PanIN, its advancement into PDAC is poorly understood [119].

For example, the lipid nuclear receptor, PPARγ, is overexpressed in human and mice PanINs. Amplification of PPARγ ligand activity via a high-fat diet markedly accelerates the transformation of PanIN to PDAC in KRAS^G12D^ mutated pancreatic epithelial cells [119]. The expression of *ALOX15B* is lost in at least 70% of prostate cancer cases [107]. Although prostatic hyperplasia is unexpectedly caused by the transgenic expression of *ALOX15B*, it failed to develop into prostatic carcinoma [120] regardless of allelic loss or complete absence of p53 [107]. Even more interestingly, there is a significant decrease in prostate intra-neoplasia and prostate cancer following transgenic expression of ALOX15B in Myc-induced prostatic adenocarcinoma [107]. It can be speculated that the hyperplasia-inducing ability as well as the potential anti-carcinogenic activity of *ALOX15B* are somewhat evocative of context-dependent pro- and anti-carcinogenic properties of Myc [121].

In the context of colorectal tumors, a comparative study of lipoxygenase products revealed no significant difference in the levels of 12-HETE, 15-HETE, and leukotriene B4 across normal, polypus, and cancerous tissues of the colon. However, a marked decrease in 13-HODE levels was an apparent shift in the metabolic hallmark of colorectal polyps and cancerous tissues [122].

The distribution of ALOX15B expression in normal lung and lung carcinomas have been reported. In benign lung tumors, immunostaining of ALOX15B was solely detected in PPARγ-expressing type-II pneumocytes. Although there were no experiments regarding co-localization of both ALOX15B and PPARγ in type-II pneumocytes, their expressions in a significant proportion of type-II pneumocytes points out a possible PPARγ-ALOX15B signaling role in modulating differentiation and proliferation of these cells [123]. Out of 160 lung carcinomas, *ALOX15B* was variably expressed in non-small cell lung carcinomas (NSCLC) including 48% of adenocarcinomas and 63% bronchioloalveolar carcinomas. Moreover, well-differentiated NSCLC exhibited higher expression of *ALOX15B* along with a significant inverse correlation between *ALOX15B* versus tumor cell proliferation (*p* < 0.0001) and tumor grade (*p* < 0.03). These data proposes regulatory functions of *ALOX15B* in proliferation in benign lung and neoplastic lung, especially in adenocarcinomas [123].

## 6. Lipoxygenases in Primary Carcinogenesis—Putative Intersections with Infections

**Figure 8:**
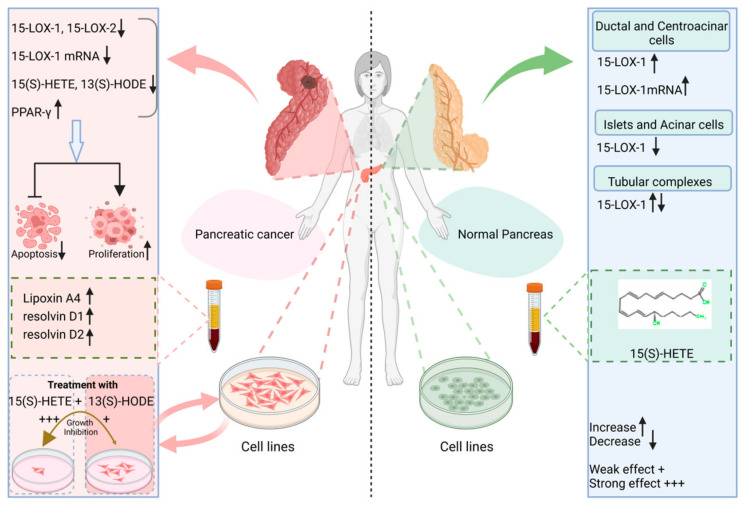
The expression pattern of ALOX15 (15-LOX-1), and ALOX15B (15-LOX-2) together with their major metabolites in normal and pancreatic tumor tissues, cell lines, and blood of patients. The levels of 15-LOX enzymes and its main products differs in healthy subjects relative to patients with pancreatic cancer. 15-LOX production and its metabolites are lost during the development of pancreatic tumors (top left). While ductal and centroacinar cells of the normal pancreas exhibit elevated levels in 15-LOX-1 protein and mRNA, tubular complexes have fluctuating expression levels of 15-LOX-1, and pancreatic islets demonstrate absence or weak expression of 15-LOX-1 (top right). Downregulation of 15-LOX-1 and 15-LOX-2 is associated with resistance in apoptosis following elevation in levels of PPARγ. Interestingly, 15-LOX-dependent bioactive lipids linked to inflammatory resolution are present in the serum of patients with pancreatic cancer relative to that of healthy individuals. Marked tumor growth inhibition is affected upon treatment of pancreatic cell lines (Mia PaCa2 and S2-O13) with low concentration of 15(S)-HETE. Similar observations are read for 13(S)-HODE but to a lesser extent and at a higher concentration (bottom left).

It is evident that certain lipoxygenases can influence carcinogenesis through multiple mechanisms (some of them are shown in Figure 8):

1. Catalyzing the activation of pro-carcinogens like aflatoxins.
2. Disruption of interferon signaling, which ordinarily promotes the identification and elimination of cells with DNA damage, thereby pre-emptively targeting potential early-stage cancerous cells.
3. Enhancing the invasiveness of existing cancerous cells by helping them evade immune surveillance.
4. Boosting the resistance of cancer cells to specific anti-cancer drugs.

These mechanisms are largely derived from the well-established roles of ALOX15, ALOX5 and its associated protein, FLAP. In the same vein, these lipoxygenases have emerging significance in the action of certain opportunistic bacteria, in several cancer-related infections.

Oxidative reactions involving eicosanoids play important roles in cancer development [124], emphasizing the apparent involvement of lipoxygenase isoforms in carcinogenesis [125]. Both platelet-type ALOX12 and ALOX5 have been associated with promoting carcinogenesis. ALOX15 and ALOX15B on the other hand majorly exhibit anti-carcinogenic properties, even though various studies demonstrate their dual roles in regulating tumor development [115,126,127].

A number of excellent reviews have presented a summary of published data on eicosanoids in cancer [128]. A huge body of evidence highlights upregulation of PGE2 in tumors due to overexpression of cyclooxygenases COX2, and COX1 in certain renal cancers [129], whereas PGI2 is anti-carcinogenic in mouse models of tobacco smoke-induced lung cancer [130].

The implication of bacteria and viruses in cancer progression has gained significant attention, with the tumorigenic function of *Helicobacter pylori* in gastric cancer being a focal point [131]. One common bacterial-mediated inflammatory infection is periodontitis. Studies have positively correlated periodontitis with the risk of developing cancers in the pancreas, breast, buccal cavity and the lung [132,133]. Out of several bacteria currently known to contribute to tumor progression, *Fusobacterium nucleatum* has only been linked to colorectal and esophageal malignancies. *F. nucleatum* primarily exists in the normal flora of the oral cavity. During pathogenesis, the bacteria should disseminate from the oral cavity to the colorectal tissues through the gastrointestinal tract [134]. Interestingly, a recent study suggests the colonization of colorectal tissues via the hematogenous route, explaining that the inhabitation of *F. nucleatum* takes place during angiogenesis in tumor tissues, facilitating bacterial trafficking from the buccal cavity following upregulation of Gal-GalNAc lectin—a biomarker of colon carcinogenesis [135]. Notably, colonization of *F. nucleatum* is not limited only to colorectal tumors, but is also found in breast cancers as well as metastasis to the lung via detection of Gal-GalNAc lectin [135].

Furthermore, the gut microflora may play a significant role in influencing the effectiveness of radiotherapy and chemotherapy in treating colonic cancers. However, to draw concrete conclusions in this area of research is a challenging task, as the correlations between microbiome imbalances and treatment efficacy are generally subtle [136].

## 7. The Oncogenic Role of Human ALOX

Lipoxygenases have been heavily implicated in oncological diseases via their oxidation reactions of long chain PUFAs and downstream eicosanoid signaling pathways [124,125]. However, the biological roles of lipoxygenase isoforms in cellular proliferation and tumor development are very complicated and experimental results have long been met with certain criticisms. Platelet-type ALOX12 and ALOX5 have been widely studied to induce carcinogenesis while ALOX15 and ALOX15B exhibit anti-carcinogenic activities although several studies demonstrate dual roles in regulating tumor development [115,126,127]. Epigenetic modification through hypermethylation of *ALOX15* promoter increases prostatic intraepithelial neoplasia. On the other hand, 13-HODE—the significant linoleic oxygenation product of ALOX15 and to a lesser extent ALOX15B—exhibits anti-apoptotic activities in colorectal cancer and hence, demonstrates anti-tumorigenic properties [137]. This proposes that the expression patterns and biological roles of lipoxygenase isoforms are not uniform across cancer subtypes.

While ALOX5 inhibition triggers marked apoptosis in human prostate cancer cells [138], it is overexpressed in prostate adenocarcinoma, PDAC [139], hepatocellular carcinoma [140], and high-grade astrocytomas [141]. Moreover, overexpression of ALOX5 in pancreatic cancer tissues has been associated with lymph node metastasis and TNM stage [97]. Expression of ALOX5 steadily increases with the progression of human Hepatocellular Carcinoma in HepG2 cells and- inhibition of ALOX5 via zileuton administration decreased cell viability and induced apoptosis in HepG2 cells [142].

Both ALOX15 and ALOX15B isoforms have lower expression levels and activities in the cancerous tissues of the pancreas, breast [143], prostate [144,145,146] and lungs [123] than in their respective normal tissues. In the pathogenesis of pituitary adenomas, the expression of both ALOX15 and ALOX15B as well as their metabolites 15-(S)-HETE, 13-(S)-HODE are significantly elevated respectively. The pro-carcinogenic role of ALOX15 isoforms was highlighted following an increase in expression and activity in pituitary adenomas with larger tumor size and a higher degree of invasion [147]. Similar to early events in the development of pancreatic cancers, expression of the *ALOX15* gene is significantly downregulated in human colorectal cancers [148,149]. Few studies stating otherwise have demonstrated significant overexpression of ALOX15 and its metabolite in human colorectal cancer epithelial cells compared to normal tissue [150]. The treatment of two human colorectal adenocarcinoma cell lines, HT-29 and DLD-1, with celecoxib increases the protein expression levels of ALOX15 by 1.5 and 2-fold respectively, while significantly increasing apoptotic rate by 2-fold compared with control cells [151]. Human colorectal adenomas and carcinomas are characterized by decreased expression of ALOX15 and low levels of 13-compared to normal mucosa [152]. Treatment of Caco-2 cells with 13-HODE led to decreased cell proliferation [153]. Interestingly, the same study noted that *ALOX15* expression was almost uniformin adenoma tissues (lower staining intensity in neoplastic epithelia and more intense staining in inflammatory regions) while the expression of ALOX15 in the healthy control tissue was for the most part restricted to colonic mucosal epithelium [153]. It is therefore necessary to rationalize that during malignant transformation, colonic cells mask the apoptotic functions of ALOX15 due to the expression of ALOX15 and that expression of ALOX15 should be localized and strictly confined regions in order to fully observe its biological activity.

On the molecular level, the products of ALOX activities affect many intracellular targets. One of the key intracellular targets—peroxisome proliferator-activated receptor (PPARγ)—is activated by eicosanoids [154]. Natural ligands of PPAR-γ include free PUFAs, flavonols, glutamine, butyrate, and phthalates. Of particular interest are PGJ2, 9-HODE, 13-HODE, 15-HETE, LTE4, and some specialized pro-resolving mediators (SPMs). PPARγ agonists regulate NF-kB-dependent inflammation by increasing levels of lkBα, an NF-kB inhibitor in mouse cystic fibrosis biliary epithelium [155]. Long chain PUFAs like EPA (eicosapentaenoic acid) and DHA (docosahexaenoic acid) stand out as potent PPARγ-dependent effectors that inhibit pro-tumor cytokines such as IL-6 and IL-8 (as reviewed in [156]), and indeed EPA upregulates PPARγ which in turn facilitates the differentiation of T-regulator cells [157].

At the transcriptional level, interleukin-4 induces the expression of *ALOX15* [158]. Mechanistically, interleukin-4 receptor activation triggers activation of STAT6 in both the colorectal Caco-2 and lung A549 cell lines. Following activation, STAT6 translocates to the nucleus, directly binds and activates genes associated with IL-4, *ALOX15* among others [159]. In A549 cells, however, activation of the ALOX15 gene by interleukin-4 was due to heightened activity of the Creb-Binding Protein’s histone acetyltransferase responsible for acetylating both STAT-6 and nuclear histones in human lung adenocarcinoma cells [160]. Additionally, the constitutive expression of ALOX15 in human macrophages significantly increases following treatment with interleukin 4 and 13 [161,162]. This leads to elevated levels of *ALOX15* mRNA and protein, which in turn boosts the production of one of its primary product, 15-HETE [163]. Similarly, a marked increase in *ALOX15* expression occurred following stimulation of monocytes with interleukin-13 [161]. Likewise, in glioblastoma cells, interleukin-13-induced expression of *ALOX15* led to the activation of PPAR-γ and the triggering of apoptosis. Furthermore, downregulation of PPAR-γ by the ALOX15 product 13-S-HODE sensitizes apoptotic signaling pathway in colorectal cancer cells [137]. Notably, STAT6 further boosts the transcription of *PPAR-γ*, underscoring the synergistic role of ALOX15, interleukin-13, and PPAR-γ in modulating cellular growth and invasion [16].

It is necessary, however, to differentiate analogous processes between cancer cells and immune cells since extracellular cues may affect tumor growth in opposite directions [164]. When pancreatic tumor cells are treated with ALOX substrates (PUFAs), there is a notable suppression of tumor cell growth [165]. A preliminary report demonstrating lipoxygenase derivatives of arachidonic acid showed that, relative to healthy subjects, patients with pancreatic adenocarcinoma and chronic pancreatitis have 3–8-fold higher levels of 5-, 12-, and 15-HETEs, and also stating that there are no marked differences in HETE levels in different TNM stages of pancreatic cancer [166]. While examining the influence of ALOX15 on pathogenic angiogenesis, ALOX15 promotes the turnover of HIF-1α and reduces VEGF expression. Inhibiting ALOX15 counteracts both effects. This discovery offers fresh insights into the regulatory mechanisms of HIF-1α [167].

In pancreatic cancer, ALOX15 is known to be lost in islets and pancreatic intraepithelial lesions but strongly expressed in normal acinar and ductal cells [126]. Upregulation of *ALOX5* is implicated in all grades of human pancreatic intraepithelial lesions, and initial development of pancreatic cancer in EL-KRAS mice and N-nitroso-bis(2-oxopropyl) amine-treated hamsters [168,169]. Findings from other research works show that overexpression of ALOX15 in pancreatic cancer cells and inhibition of ALOX5 and platelet-type 12-lipoxygenase exert anti-tumorigenic effects [126].

Important for our review is the classification of tumors according to the degree of infiltration by immune cells. For example, glioblastoma is a classical cold tumor which prefer to grow and disseminate in a stealth mode secreting immunosuppressive cues, whereas bladder cancers usually belong to the hot type which employs another strategy that relies on creating a flare-up for the host immune system via the build-up of excessive chronic inflammation that is detrimental to the incoming anti-cancer immune cells. Other biolipid mediators like prostaglandins, contribute to the immunosuppressive environment of cancer tumors [170]. Thus, the functions of oxylipin signaling are rather context-dependent, and are more difficult to predict in intermediate cases like PDAC that relies mostly on reprogramming fibroblasts into TAMs, which creates a tumor friendly microenvironment.

## 8. The Anti-Tumorigenic Function of ALOXs

Because resistance to apoptosis is a hallmark of many infections and tumors, non-apoptotic cell death gains extensive recognition in tumor therapy and disease treatment [171]. Ferroptosis is one such non-apoptotic modes of cell death. It is described as an iron-dependent controlled necrosis driven by lipid peroxidation and reactive oxygen species (Lipid-ROS) [172]. However, cancer cells have developed specific survival pathways to evade this cell death mechanism by detoxifying Lipid-ROS via several pathways including augmentation of Gpx4 glutathione peroxidase activity, which uses glutathione (GSH) as a co-factor [173].

**Figure 9:**
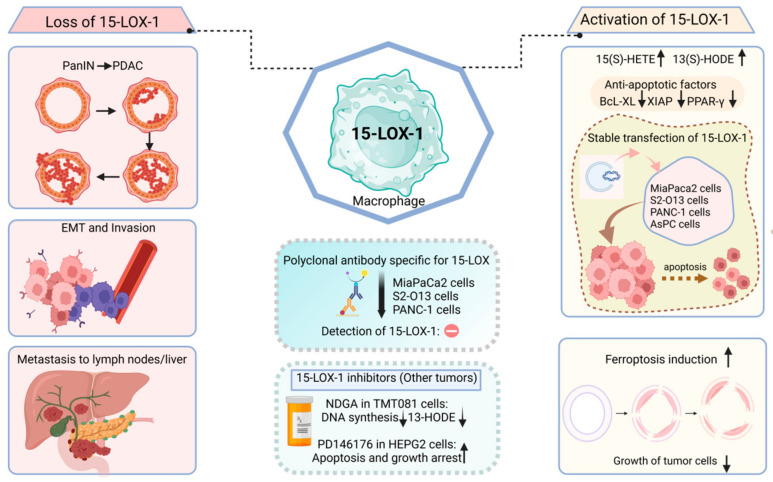
The effect of ALOX15 (15-LOX) activity and their main metabolites in pancreatic cancer development. The activity of ALOX15 (15-LOX-1) and ALOX15B (15-LOX-2) can influence pancreatic cancer invasion and metastasis through a number of pathways. MiaPaca2 cells, including a number of pancreatic cancer cell lines exhibit no expression of 15-LOX-1. Overexpression of 15-LOX-1 in PANC-1, MiaPaCa2, AsPC, and S2-O13 cells leads to downregulation of anti-apoptotic proteins like XIAP, inhibiting tumor growth. As a ferroptosis driver gene, 15-LOX has been proposed to somewhat contribute to tumor cell death by ferroptosis. Acting as a tumor suppressor gene in pancreatic tumors, downregulation of 15-LOX-1 during early development of this tumor contributes to progressive development of pancreatic intraepithelial lesions to pancreatic ductal adenocarcinoma, invasion and metastasis into the lymph nodes and liver.

There is mounting evidence that autophagy plays a context-dependent role in facilitating ferroptosis [174], despite the fact that ferroptosis was formerly thought to be an autophagy-independent form of cell death [175]. Recent studies have identified the nuclear receptor coactivator 4 (NCOA4) protein as a cargo receptor for ferritinophagy, which facilitates an increase of the intracellular iron pool (IIP), thereby contributing to Lipid-ROS generation and cell death [176,177] (Figure 9).

In this respect, lipoxygenases have been implicated as key mediators in ferroptosis. Although lipoxygenases are not crucial for ferroptosis to occur, they aid to its onset by contributing to the pool of lipid hydroperoxides that enhance lipid oxidation [178]. A number of studies have shown the relationship between ferroptosis and ALOX5. ALOX5 promotes lipid peroxidation of PUFAs containing phospholipids which are one of the central players in driving ferroptosis [179]. As a novel target in melanoma drug development, ALOX5 has been described to promote autophagy-dependent ferroptosis by activating the AMP-activated protein kinase (AMPK) pathway in vitro and in vivo [180]. Via the AMPK signaling pathway, the upregulation of *ALOX5* mRNA and protein levels is associated with the depletion of GSH, accumulation of intracellular iron, malondialdehyde production, lipid peroxidation, and marked growth inhibition. Indeed, the silencing of *ALOX5* significantly reduced mitochondrial damage and increased the number of autophagosomes in erastin-treated melanoma cells (A375, A-875) demonstrating the occurrence of iron-dependent death and autophagy in the cells [181]. In a more recent study, overexpression of *ALOX5* sensitizes bladder cancer cells to ferroptosis. On the contrary, *ALOX5* knockout contributes to bladder cancer development by mediating escape from ferroptosis. Notably, loss of ALOX was regulated by EGR1 at the transcriptional level [99]. ALOX15 and their polyunsaturated substrates can promote ferroptosis [182,183,184,185,186] and tumor ferroptosis have been shown to be enhanced by ALOX-catalyzed lipid peroxidation in cellular membranes after induction by erastin and RSL3 [187]. Binding of ferrostatin to ALOX5/PEBP1 (phosphoethanolamine binding protein 1) effectively inhibits the enzyme and the production of its metabolite HpETE-PE, which ultimately prevents ferroptosis [188]. The action of lipoxygenase enzyme has been exploited in tumor-killing nanoreactors to induce ferroptosis and anti-tumor immunity in order to ultimately enhance the therapeutic advantage of radiofrequency ablation in preclinical models [189].

### 8.1. Hypoxia, Neoangiogenesis

In anoxia, lipoxygenase reactions are expected to cease as hypoxia induces significant changes in gene expression, with hypoxia-inducible factor (HIF) playing a well-recognized role in these alterations. It is conceivable that ALOX enzymes might have non-enzymatic functions influenced by these changes. The response to hypoxia is crucial for tumor growth, and ALOX enzymes may have significant roles in this process. Specifically, ALOX15 facilitates ubiquination and degradation of HIF-1α and reduces the expression of Vascular Endothelial Growth Factor (VEGF). Conversely, inhibiting ALOX15 has the opposite effect. This discovery provides new insights into the regulatory mechanisms of HIF-1α [167]. Redox equilibrium plays a pivotal role in shaping gene expression, thereby influencing the phenotypic characteristics of mammalian cells. For example, overexpression of *ALOX15* in the U-937 human histiocytic lymphoma cell line results in altered gene expression patterns, though the full phenotypic implications of these changes are yet to be fully understood. Given these observations, it is worth considering whether lipoxygenases might function in a manner akin to oxygen sensors.

### 8.2. P53, the ALOX Gene Family and Micro-RNAs

Numerous recent studies point to the ambivalent role of ALOX proteins in tumorigenesis. On one hand, ALOX proteins promote tumorigenesis by increasing the ROS production and on the other hand, certain members of the ALOX family exhibit anti-tumorigenic features. In this respect, it is important to mention the association of ALOX proteins with one of the major tumor suppressors, p53. The p53 protein is operating mostly as a regulator of transcription of protein-coding and non-coding genes whose products participate in cell cycle arrest and apoptosis [190,191,192]. The activity and protein stability of p53 is controlled by an E3 ligase, Mdm2 [193,194]. Thus, many attempts have been made to develop therapeutic inhibitors of Mdm2, and some of them are currently in clinical trials [195,196,197]. Accordingly, given that the link between p53 and ferroptosis has been firmly established [198,199], several reports investigated the relations between p53 and ALOX proteins during tumorigenesis. For example, by using unbiased whole-genome ChIP-seq analysis, p53 was found to bind the G-intronic region of *ALOX5*. In agreement with the mechanism of p53 activation, the expression of *ALOX5* was induced by genotoxic stress mediated by genotoxic drugs, actinomycin D or etoposide [200]. Notably, in the same study, ALOX5 directly binds and blunts the transcriptional activity of p53, suggesting that a negative feedback mechanism exists to restrict the activity of p53 in tumor cells. In line with this notion is the observation that ALOX5 counteracts genotoxic drug-induced apoptosis in cancer cells by interfering with the activation of proapoptotic genes regulated by p53 [201]. Mechanistically, ALOX5 interferes with p53 functions by inhibiting relocalization of the tumor suppressor gene into PML nuclear bodies. When ALOX5 is inhibited using zileuton, it results in reduced cell viability and increased apoptosis in HepG2 cells [107].

In drastic contrast to its role in tumor cells, ALOX5 in non-transformed cells promotes the p53/p21-induced growth arrest caused by forced expression of oncogenic Ras [202]. Unfortunately, the effect of the p53/ALOX5 axis on ferroptosis was not examined in these studies. Furthermore, p53 activates expression of another member of the *ALOX* gene family, *ALOX15B.* Inhibition of the activity of iron anti-porter, SLC7A11, leads to the induction of ferroptosis in bladder cancer cell [203].

*ALOX15B* is significantly silenced in bladder cancer, and SCL7A11 is reported to induce tumor growth through suppression of ferroptosis. In providing insights to the mechanistic development of bladder cancer, p53 activates *ALOX15B* through the blocking of cystine transporter SLC7A11 to induce ferroptosis [203]. The effects of ALOX15B and p53/SLC7A11 on bladder cancer cells were evaluated by in vitro and in vivo experiments. These experiments revealed that knockdown of *ALOX15B* promoted bladder cancer cell growth, which was also found to protect bladder cancer cells from p53-induced ferroptosis. Finally, p53 activated *ALOX15B* activity by suppressing SLC7A11 in bladder cancer [203].

Another layer of complexity to the p53-ALOX gene network is provided by variability of expression of various micro-RNAs. p53 is known to regulate a plethora of non-coding RNAs thereby possibly targeting the mediators of ferroptosis [204]. On the other hand, p53 itself can be the target of microRNAs activated via ALOX-associated pathways. For example, miR-660—which targets ALOX15B in cervical cancer cells [205]—also attenuates the expression of Mdm2 in lung cancer cells [87], thereby releasing p53 from the negative regulation and hence, augmenting the level of *ALOX15B* expression. In contrast, miR-125b, which inhibits p53 translation [206] promotes ferroptosis in gastric cancer cells [207]. A plausible hypothesis explaining these contradictory results may be that ferroptosis acts as a means to control just appeared malignant cells through the death of cells with increased ROS production.

ALOX12 is required for p53-mediated tumor suppression through a distinct ferroptosis pathway [208]. Indeed, a strong piece of evidence was presented in this important work by [208], with the authors observing that p53 activation in xenografted tumors disconnects ferroptosis from GPX4 glutathione peroxidase and thus makes cells more sensitive to ROS. In that model ALOX12 was found to be important for p53 action, and it was demonstrated that the blocking of ALOX12 reduces p53-mediated ferroptosis activated by ROS. A similar hypothesis has been proposed in relation to ALOX12 inhibition in tumor cells from cervical squamous cell carcinoma, head and neck squamous cell carcinoma, esophageal squamous cell carcinoma and acute myeloid leukemia. This whole pathway was independent of ACSL4 which is required for ferroptosis upon GPX4 inhibition [208]. The actual mechanisms behind this interesting discovery remain unclear, thus, it is difficult to stratify cancer patients according to their p53 status for example, and it is uncertain how it affects the action of this axis especially with respect to radio- and chemotherapy.

### 8.3. Lipoxygenases and Immune Suppression in Tumor Growth

A good number of studies have evaluated the role of sphingosine-1 phosphate (S1P) in lymphomas. In detailing the mechanism of sphingosine-1-phosphate (S1P) in obesity-lymphomagenesis, a recent study demonstrated that up-regulated S1P-S1P receptors 1/2- YAP signaling mediates the aggressive nature of obesity-lymphoma by inducing cell proliferation and migration. More importantly, S1P-ALOX15 signaling mediates polarization of macrophages towards tumor-associated macrophages, establishing an immunosuppressive microenvironment [209]. In a separate study, both ALOX15 and ALOX15B isoforms were identified in normal mammary epithelial cells as well as in vascular endothelial cells, and the expression of both isoforms was considerably diminished in breast tumor tissues [143]. Trichostatin A-induced sensitization of apoptosis and cell-cycle arrest in breast cancer cell lines MCF-7 and MB-MDA-231 have been associated with *ALOX15* induction and elevation of 13-S-HODE production [115,126,127].

Among several mono- and polyunsaturated fatty acids, linoleic acid could be important for triple negative breast cancers overexpressing fatty acid binding protein-7. MDA-MB-231 cells overexpressing this protein undergo linoleic-acid induced cell death, evidenced by low levels of 13-HODE—a pro carcinogenic metabolite in the setting of breast tumors [210].

Human ALOX5, ALOX15, and ALOX12, coexist in the kidney—but they exhibit opposite trends and their balance switches during carcinogenesis. There are high levels of ALOX15 and low levels of ALOX5 and ALOX12 at the onset of cancer, which reverses with increasing tumor grade and progressing stage [211].

Research by Kelavkar and his team revealed that epigenetic alterations—specifically hypermethylation of the *ALOX15* promoter—amplify prostatic intraepithelial neoplasia. Interestingly, as a minor linoleic oxygenation byproduct of both ALOX15 and ALOX15B, 12-HODE displays anti-apoptotic actions in colorectal cancer, suggesting its potential anti-tumorigenic properties [212].

### 8.4. Human ALOXes and Tumor-Associated Inflammation

The patho-physiological function of lipoxygenase is not limited to the mere formation of bioactive lipids, but the effects of the catalytic activity of these enzymes goes a long way to influence the cellular redox homeostasis. The cellular redox state of cells is critical in defining and regulating the expression pattern of genes which consequently modifies the phenotypic profile of mammalian cells [213]. The eicosanoids and docosanoids involved in the resolution of inflammation are normally metabolized from three major PUFA substrates: arachidonic acid, docosahexaenoic acid and eicosapentaenoic acid. These mediators are as a result of concerted signaling between ALOX5, ALOX15, ALOX15B, and ALOX12. For example, 15S-hydroperoxyeicosatetraenoic acid may be further oxidized by ALOX5 into lipoxins that resolve acute inflammation [85,86].

Human ALOX15 demonstrates both pro-inflammatory and anti-inflammatory activities in varying inflammation models. Mounting evidence uncovers the critical role of ALOX15 during inflammation resolution, where their derived products (15-HETE and 13-HODE) exert anti-inflammatory effects through nuclear and cell-surface receptors along with intervention of pattern recognition receptors [214]. Similarly, following the creation of transgenic mice expressing human *ALOX15* under the regulation of activating protein 2 promoter (aP2-ALOX15 mouse), systematic blocking of ALOX15 gene escalated inflammatory indicators whereas its overexpression minimized inflammation in a hind-paw edema model. However, in a dextran sodium sulfate colitis model, overexpression of *ALOX15* hardly impacted the severity of inflammatory indicators even though systematic blocking of the gene did protect female mice from colitis induced by dextran sodium sulfate. Thus the implication of *ALOX15* in the pathogenesis of inflammatory diseases remains variable and inflammation model-dependent [215].

In the pathogenesis of asthma, a pro-inflammatory MAPK/ERK signaling pathway is activated to induce hypersecretion of mucus, following binding of abundant epithelial ALOX15 to phosphatidylethanolamine-binding protein in human asthmatic epithelial cells in vivo [216].

ALOX5 and COX-2-derived eicosanoids play critical functions in the development of colorectal cancer, thusthe dual targeting of these enzymes is a plausible strategy [217]. Inhibition of ALOX5 in tumor cells leads to varied impacts on gene expression profiles and cellular functions depending on the cell type. Notably, genes associated with cytoskeletal organization, cell adhesion, and formation of extracellular matrix undergo significant changes. Consequently, such alterations in gene expression influence cell motility and proliferation. For instance, the consequence of gene expression was studied following knock-out of ALOX5 in multiple cancer cell lines: TGF-2 expression was elevated in HCT-116 cells, expression levels of MCP-1 and platelet-derived growth factor were reduced in U-2 OS cells. Also, the knockout of *ALOX5* has an effect on cell motility and proliferation. Intriguingly, pharmacological inhibition of ALOX5 only partially mimicked the knockout, implying that there may be noncanonical roles at play [218]. Lack of migrated natural killer T cells facilitates development of pancreatic cancer and their presence modulate tumor associated macrophages (M2) through ALOX5 and mPGES-1 [219].

The role of lipoxygenase products in T cell function can be summarized as follows:

1. 15-HETE enhances T cell proliferation, particularly promoting Th1 cell activity.
2. 12-HETE facilitates chemotaxis, although it is more specifically a chemoattractant for neutrophils, and the receptors for this activity are not yet identified.
3. Lipoxin A4 (LXA4), through the BLT1 receptor, stimulates the production of cytokines.
4. Leukotriene B4 (LTB4), also acting through the BLT1 receptor, encourages T cell homing.
5. Cysteinyl leukotrienes D4 (CysLTD4) and E4 (CysLTE4), interacting with the CysLT1 receptor, are involved in inducing Th2 cell differentiation [157].
6. Metabolites of ALOX5 and ALOX12 are elevated in human colon adenoma tissue and serve as therapeutic targets for colorectal cancer chemoprevention [220].

In both pancreatic and colon cancers, a substantial body of research supports the role of inflammation in their pathogenesis. The anti-inflammatory properties of special pro-resolving mediators (SPMs) counteract cytokines-promoting tumorigenesis [112]. Recent research indicates that SPMs mitigate inflammation in the β-cells of the pancreas and the enterocytes of the colon—but the exact etiology and molecular consequences of how immune responses in pancreatic and colon cancers are linked remains unclear [221]. Peripheral levels of lipoxygenase-derived immunoresolvents (lipoxins A4, B4, and resolvins D1, D2) are 2–10 fold higher in patients with pancreatic cancer relative to healthy subjects [222]. Taking into consideration the unrestrained inflammation characteristics of pancreatic tumorigenesis, it is logical to reason that significantly upregulated levels of these immunoresolvents is probably an inhibitory feedback to keep this inflammatory processes at bay [222]. While the role of ALOX15 and ALOX15B in tumor-associated macrophages (TAMs) is not yet fully understood, numerous ALOX15/B metabolites, particularly resolvins and lipoxins, exhibit anti-tumorigenic properties [223].

In the pathogenesis of pituitary adenomas, the expression of both ALOX15 and ALOX15B as well as their metabolites 15-(S)-HETE, 13-(S)-HODE are significantly elevated. This pro-carcinogenic role of ALOX15 isoforms was highlighted following an increase in expression and activity in pituitary adenomas with larger tumor size and higher degree of invasion [147]. Expression of *ALOX15B* mRNA and protein is lost in esophageal cancers and upregulation of *ALOX15B* during a COX-2 inhibitor, NS398, treatment is associated with reduced cancer cell survival and proliferation [224].

The ALOX15-derived metabolite 13-(S)-HODE has been shown to augment the MAP kinase signaling pathway and diminishes PPARγ in colorectal cancer cells. Opposingly, there is a loss and increase in the expressions of *ALOX15B* and *ALOX15*, respectively, in prostate tumor tissues. Specifically, ALOX15-derived 13-HODE and ALOX15B-derived 15-HETE upregulated and downregulated the MAP kinase signaling pathway, respectively, in prostate cancer PC3 cells. Ultimately, 15-HETE reduced PPARγ phosphorylation, whereas 13-HODE produced its decrease. Hence, ALOX15 metabolites have contrasting effects on modulating the MAP kinase pathway and downstream factors such as PPARγ. Furthermore, the role of EGF and EGFR in these mechanisms is a subject of interest, as is the role of IGF-1, which is known to activate both MAPK and Akt pathways [225].

## 9. Inhibitors of ALOX in Anti-Cancer Therapies

The use of small molecule inhibitors to target complementary pathways have gained interest in cancer therapy [226]. Epidermal growth factor receptor (EGFR), ALOX5 and COX-2 are overexpressed in PDAC. Gefitinib (EFGR inhibitor) and licofelone (dual COX-ALOX5 inhibitor) significantly inhibited the incidence of PDAC in genetically engineered mice. Synergistic treatment led to the complete inhibition of PDAC [227]. In vitro and in vivo experiments respectively in MiaPaCa-2, AsPC-1 human pancreatic cancer cell lines, and athymic mice xenograft models, show that inhibitors of ALOX5 (Rev-5901) and ALOX12 (baicalein) induce growth inhibition while activating apoptosis via the mitochondrial pathway [228]. Furthermore, these same two inhibitors in addition to general lipoxygenase inhibitor (NDGA) induced apoptosis as well as noticeable altered cellular morphological changes concordantly with increased activity of carbonic anhydrase in four pancreatic cancer cells (Capan2, MiaPaca2, HPAF and PANC-1) [229]. In enzalutamide-resistant prostate cancer cells, the inhibition of ALOX5 interferes with c-Myc signaling, killing cells by enhancing caspase-mediated apoptosis [230]. Triple targeting of ALOX5, COX-2 and double mutant EGFR by novel quinazolinone tethered phenyl urea derivatives demonstrate anti-inflammatory and anti-cancer activities in numerous cancer cell lines, not limited to BT-459 breast cancer cell line [231]. The invasive and metastatic role of ALOX5 in the progression of pancreatic cancer have been demonstrated both in vitro and in vivo using an approved ALOX5 inhibitor, Zileuton. The study discovered a signification correlation between increased levels of ALOX5 and poor survival, perhaps due to the activation of JAK/STAT signaling in macrophages which reprograms macrophages to the M2 phenotype. Inhibition of ALOX5 by low-dose Zileuton led to the inhibition of invasion and metastasis in PANC-1 pancreatic cancer cells, and mitigated the M2-like phenotype through the JAK/STAT pathway in macrophages [232]. Following zileuton treatment in nude mouse model of in situ transplantation tumor of pancreatic cancer, mice exhibited improved survival and reduced liver metastasis. These findings show that ALOX5 regulates TAMs polarization through the JAK/STAT pathway, promoting invasion and metastasis in pancreatic cancer [232].

In the evaluation of lipoxygenases as targets in malignant pleural mesothelioma cell lines (NCI-H2052, NCI-H2452, and MSTO-211H), expression of *ALOX12* and *ALOX5* is highly upregulated in the majority of the samples. Inhibition with baicalein was effective in all three cell lines at low concentrations with IC_50_ between 9.6µM and 20.7µM respectively. Thus, baicalein holds promise in therapy for malignant pleural mesothelioma [233]. Similar studies have demonstrated the use of both ALOX12 and ALOX5 inhibitors in pancreatic cancer cells PANC-1 and Capan2, which were shown to express ALOX5 and ALOX12. Along with their respective metabolites (5-HETE and 12-HETE) and substrates (arachidonic and linoleic acids), they all stimulate pancreatic cancer cell proliferation, and there was direct reversal of inhibitor-induced grown inhibition by both 5-HETE and 12-HETE [234].

Blocking of arachidonic acid metabolism via the use of NDGA (ALOX inhibitor) or ETYA (a dual COX and ALOX inhibitor), inhibits cell proliferation, matrix metalloproteinase activity and invasion in head and neck cancers [235]. Endothelial cell-specific overexpression of ALOX15 in Lewis Lung Carcinoma induces necrosis and apoptosis in primary and metastatic tumors by elevating the expression of PPARγ and P21 in neighboring cancer cells in mice [236]. Combination of ALOX12 inhibitors (baicalein and BMD122) and radiation therapy have synergistic inhibitory effects on the growth of LNCaP and PC-3 prostate cancer cells as well as prostate cancer xenografts in SCID mice [237]. In a syngeneic tumor model of non-small cell lung cancer and melanoma, ALOX12 inhibition was showed to mitigate the effect of pre-radiation on the growth of pulmonary tumor nodules [238].

Targeting of sphingosine-1 phosphate (S1P) is now of growing interest following the contribution of ALOX15-S1P signaling in exacerbating lymphomagenesis via polarization of normal macrophages towards TAMs creating immunosuppressive microenvironment in obesity-related lymphomas [209]. Interestingly, treatment of obesity-lymphoma mice with a natural phenol (resveratrol) revealed marked effects of anti-lymphomagenesis through downregulation of S1P-YAP axis and reprogramming macrophage polarization from M2 to M1 phenotype [209].

In experimental cell lines, 13-HODE inhibited proliferation of MCF7 and MDA-MB-231 breast cancer cell lines in a time/dose dependent manner. It was demonstrated that mounting levels of 13-HODE were associated with cells accumulated in the G1 cell cycle phase, PPARγ downregulation, and initiation of apoptosis [239]. Additionally, 13-S-HODE has been identified to bolster doxorubicin resistance in MCF-7 cells, inducing apoptosis, cell cycle arrest, and PPAR activation. An intriguing cascade occurs where there is auto-activation of ALOX15, leading to an elevation in caspase 3/7 activity [240].

Differentiation of cells in normal tissues or quasi-differentiation processes in cancers may be modelled by cell culture with some additives like butyrate—it has the capability to amplify ALOX15 levels, thereby elevating 13-S-HODE. Moreover, treatment of undifferentiated human neuroblastoma cells with sodium butyrate (histone deacetylase inhibitor) markedly increased *ALOX15* mRNA expression [241]. In contrast, DHA curtails tumor growth and angiogenesis due to its inhibitory effect on 15-HETE synthesis [241].

Collective evidence proposes a potent pro-cancerous influence of ALOX15/ALOX15B and their reaction products in breast tumor tissues. This is primarily due to the increased levels of cAMP, phosphorylation of p38-MAPK, and DNA synthesis, all of which are pro-cancerous elements influencing spheroid formation, CREB activation, and TGF-α. This entire cascade, however, remains sensitive to the availability of lipids and free acids [88]. It is worth noting that dexamethasone, a compound frequently incorporated in oncological therapeutic regimens to ameliorate side effects, intriguingly elevates mRNA and protein levels of *ALOX15B* in monocyte-derived macrophage phenotypes, leading to production of SPMs in inflammatory cells [242].

## 10. Control of Lipoxygenation in Cancer Prevention and Treatment

Diverting the arachidonic acid metabolism towards 15-HETE can be achieved via COX-2 inhibition. In studies examining the role of ALOX15 and its metabolites across five glioblastoma cell lines (namely, U251-MG, U87-MG, U138-MG, T98G, and A172), a notable increase in 13-HODE levels was observed relative to other lipids analyzed. Using two ALOX15 inhibitors (luteolin and nordihydroguaiaretic acid) resulted in a decrease in glioblastoma growth, migration, and invasion while concurrently increasing in cell cycle arrest in the G2/M phase [243]. The effect of other PUFAs (eicosapentaenoic and γ-linoleic acids) have similarly been shown to reduce cell growth and regulate drug-resistant-ATP binding cassettes (ABC) transporters in glioblastoma cells [244].

Utilizing small molecule inhibitors to simultaneously target multiple complementary pathways has become a prominent approach in cancer treatment [226]. In the case of PDAC, there is consistent overexpression of the Epidermal Growth Factor Receptor (*EGFR*), *ALOX5*, and *COX-2* [227]. Despite the fact that there are hurdles associated with the use of tyrosine kinase inhibitors [245], the application of Gefitinib, an EGFR inhibitor, alongside Licofelone, which inhibits both COX and ALOX5, has proven to be effective in hindering PDAC carcinogenesis. This effectiveness was notably enhanced when these inhibitors were used in combination in a genetically engineered mouse model, underscoring the potential of multi-targeted therapy in cancer treatment [227].

## 11. Perspectives of ALOX15 Transgene, a Oncotherapeutic Booster

Overexpression of *ALOX15* induces p53-dependent growth arrest in human colorectal cancer cells, HCT-116 [246]. Using an adenoviral delivery system to express ALOX15 in-vitro and in vivo in colon cancer models (HCT-116, HT-29, LoVo xenografts) expression and activities of ALOX15 were restored to therapeutic levels, expression of anti-apoptotic proteins including BcL-XL, X-linked inhibitor of apoptosis protein (XIAP) were downregulated, and growth of colon cancer xenografts in vivo as well as survival of cancer cell in vitro were inhibited [247].

Furthermore, downregulation of PPAR-γ by the ALOX15 product 13-S-HODE sensitizes apoptotic signaling pathway in colorectal cancer cells [137]. These results are consistent with the works of Luo and colleagues who demonstrated that ALOX15 expression is negatively associated with infiltration of tumor-associated macrophages in cervical cancers [205].

In approximately 70% of prostate cancer cases, there is an absence of *ALOX15B* expression. Interestingly, while transgenic introduction of *ALOX15B* can cause hyperplasia, it does not lead to the development of prostate cancer, even when the p53 gene is inactive [120]. Moreover, the transgenic expression of ALOX15B in prostate cancer driven by the *Myc* gene significantly reduces both prostatic intraepithelial neoplasia and prostate cancer [107]. Functioning as in a tumor suppressor mode in the prostate, the alternatively spliced variants of ALOX15B are found to induce cell-cycle arrest and senescence in the epithelial cells of the normal human prostate. The downregulation of ALOX15B and its spliced isoforms is closely linked to unchecked cell proliferation, a key factor in the development of prostate cancer [248].

It is already known that ALOX15B is significantly downregulated in head and neck carcinoma [249]. As a candidate for radiotherapy booster in head and neck carcinoma, the levels of ALOX15B and its major metabolite 15-S-HETE increased two-fold and three-fold respectively in head and neck cancer cells following transfection with *ALOX15B* expression shuttle and 4 Gy of radiation. This upregulation of ALOX15B led to increased apoptosis enhancing the impact of radiotherapy in head and neck cancers. Overall, there was a synergistic effect between ALOX15B and radiation in head and neck cancer models [250].

In bladder cancer, p53 activates ALOX15B by inhibiting SLC7A11, leading to the induction of ferroptosis in bladder cancer cells [203]. In melanoma tumors, the blocking of ALOX5 or ALOX12-associated eicosanoid production reverses Schwann cell-dependent suppression of anti-tumor T cell activation [251]

Current clinical trials targeting malignant glioma have shown promising therapeutic benefits from using herpes simplex virus thymidine kinase (*HSV-TK*) gene therapy [252]. However, when *HSV-TK* was combined with *ALOX15* using adenoviral vectors in BT4C malignant glioma cells, there was no notable increase in tumor growth inhibition. Instead, the significant effect was observed in the altered migratory properties of the tumor cells, leading to a decreased cell survival [253].

**Figure 10:**
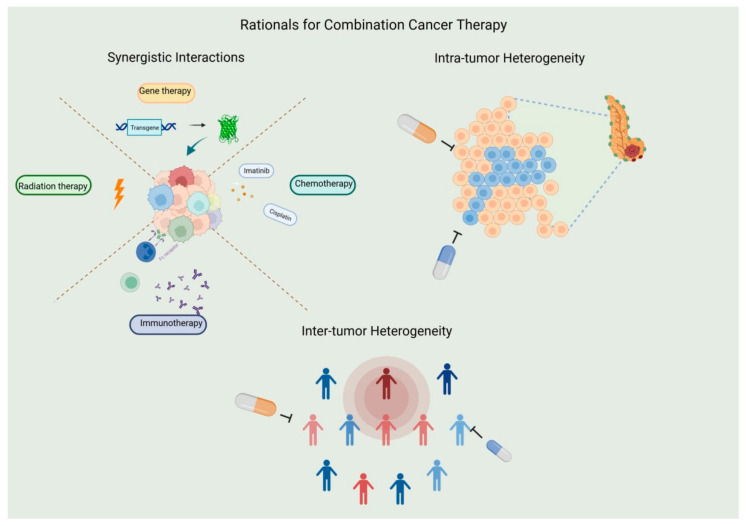
Three general rationales for combining cancer therapies: Additive or synergistic effect of multiple complementary drugs are effective in killing resistance-susceptible tumor cells. Secondly, combinations of several drugs are effective in overcoming the clonal heterogeneity developed during tumor development. This mechanism can be as simple as resistance of specific tumor cells to a specific drug but sensitive to another drug with a different mechanism of action. The same logic can also play out at the scale of patient-to-patient variability.

The actions of lipoxygenases have been efficiently harnessed in tumor-killing nanoreactors [189]. The complete elimination of large and multiple-metastatic solid tumors post radiofrequency ablation usually poses a challenge [254]. The development of cancer-debris-fueled nanoreactors encapsulating lipoxygenases constantly produce cytotoxic lipid radicals which suppress residual tumors via ferroptosis induction and priming of antitumor immunity post radiofrequency ablation [189]. This approach opens up new avenues for augmenting cancer treatments through innovative technological applications, especially if these are fine-tuned to be active in a specifical tumor milieu such as in acidosis [255]. Some of the ideas in favor for the use ALOX15B augmentation are shown in Figure 10.

## 12. Conclusions

There is a growing recognition of a dual challenge facing healthcare systems worldwide: simultaneously addressing infections and cancer. This complex scenario calls for extensive research into the interactions between pathogens and carcinogenic processes. Emerging research indicates that cystic fibrosis survivors, often exposed to *P. aeruginosa*, have an increased risk of developing colonic cancer. These intricate connections likely involve critical factors like the dysregulation of eicosanoid signaling, highlighting the need for a multi-disciplinary approach to effectively tackle both infections and cancer development.

Understanding the mechanisms that distinguish pre-cancerous tissues and benign growths from aggressive malignancies capable of distant metastasis remains a significant research gap. Focusing on differentiating these various stages is crucial for advancing development of new cancer treatments.

## Figures and Tables

**Table 1:** Kinetic Parameters of Various Lipoxygenase Isoforms.

Lipoxygenase	Optimum Temperature (°C)	Thermal Instability (°C)	pH	Vmax (µM/min)	Km (µM)	K_cat_ (s^−1^)	K_cat_/K_m_ (µM^−1^s^−1^)	References
Soybean ALOX15	35–40	>60	9.0		15 ^b^	287	19 ^b^	[12,42,43]
Human ALOX15	22, 25–30	>40	7.0, 7.5	1.03 ^a^ 4.9 ^b^	7.5 ^a^ 3 ^b^	5.3 7.8	2.0 ^a^ 2.5 ^b^	[12,44,45] [46,47]
Human ALOX15B	37	>45	8.5		3.5 ^a^	0.74 0.14	0.1 ^a^ 0.013 ^b^	[45,48,49]
LoxA	22–35, 25	>45 °C, t_1/2_ = 10 min at 50 °C	6.5, 7.5	0.226 ^b^	12 ^a^ 7 ^b^	181 28	16 ^a^ 3.8 ^b^	[43,50,51]
Human ALOX5	21, 25	>40 °C ^c^	8.0	2.56 ^a^	22.3 ^a^	0.06	0.054 ^a^	[52,53,54,55]

^a^ Arachidonic acid as substrate. ^b^ Linoleic acid as substrate. ^c^ guinea pig enzyme.

## Data Availability

Not applicable.
